# Longitudinal Trajectories of Dental Attendance in Australian Adults

**DOI:** 10.1177/00220345251315155

**Published:** 2025-03-12

**Authors:** G. Kaur, T. King, A. Karahalios, A. Singh

**Affiliations:** 1 Centre for Epidemiology and Biostatistics, Melbourne School of Population and Global Health, University of Melbourne, Melbourne, VIC, Australia; 2 Centre for Health Equity, Melbourne School of Population and Global Health, University of Melbourne, Melbourne, VIC, Australia; 3 The University of Sydney School of Dentistry, University of Sydney, Sydney, NSW, Australia

**Keywords:** socioeconomic factors, health care disparities, health equity, health services accessibility, oral health, life change events

## Abstract

Understanding how dental attendance evolves throughout life can inform targeted preventive health care policies by identifying key moments when people are more or less likely to seek dental care. Trajectory modeling of age and time trajectories takes a life course approach to understanding dental attendance, offering insights into both developmental perspectives (e.g., life stages) and structural perspectives (e.g., social position and health care systems) throughout the life course. This study used group-based trajectory modeling to identify (1) the age trajectories of dental attendance among Australian adults from young adulthood to retirement age and (2) the distinct time trajectories of dental attendance among Australian working-age adults. Data from the Household, Income and Labour Dynamics in Australia (HILDA) study was used to fit 2 trajectory models (age and time based). Age trajectories were fitted for individuals aged 15 to 64 y using dental attendance data from 3 time points: 2009, 2013, and 2017. Time trajectories were fitted for working-age adults (24–54 y) using data from 2009 to 2017 and descriptively analyzed by social characteristics. Dental attendance was classified as frequent (less than 2 y since the last visit) or infrequent (2 y or longer). Two distinct age trajectories emerged among participants (*N* = 11,189): the mostly frequent (75.1%) and declining-infrequent group (24.9%). A sharp decline in the probability of being frequent attendees was observed between 15 and 20 y in a quarter of the population with no subsequent change. Four time trajectories were identified (*n* = 7,033): consistently frequent (37.8%), consistently infrequent (8.9%), increasing attendance (22.2%), and declining attendance (31%). Descriptive analysis showed that age and social inequalities were evident in the trajectories. The findings emphasize the need for preventive health care policies that account for life-stage dynamics and their impact on attendance behaviors, in addition to improving structural factors.

## Introduction

A favorable pattern of dental attendance, characterized by regular preventive dental visits with the same dental professional, improves oral health and enhances quality of life (Ellershaw et al. 2011). In addition, the longer regular attendance is maintained, the better the oral health (Thomson et al. 2010). However, approximately half of adults in Western countries visit a dentist regularly (Reda, Krois, et al. 2018).

Social inequalities exist in oral health outcomes and in accessing and using oral health services (World Health Organization 2022). Life course approaches explain how various social structures and exposures at different developmental stages shape health, highlighting the underlying causes of health inequalities (Jones et al. 2019). For dental attendance, the structural perspective of the life course approach emphasizes the roles of social position, economic resources, health care systems, and policies in shaping people’s access to and utilization of dental care (Farmer et al. 2016). The developmental perspective emphasizes the role of life stages, critical life events and transitions (such as entering the workforce or retiring), in shaping dental attendance (Crall and Forrest 2018).

In Australia, barriers to accessing oral health care include affordability, time constraints, dental anxiety, limited public dental care, and extended waiting times (Australian Institute of Health and Welfare 2023). Sustained and consistent oral health care policies implemented over a substantial duration are lacking, and most policies focus only on younger or older age groups rather than working-age individuals (Duckett et al. 2019). Working-age people remain at risk and ignored from policy. A life course perspective is needed to better understand the variations in dental attendance over time, in particular how significant life developments such as transitioning to working life and consequent competing demands affect the resources needed for favorable dental attendance. These resources, including flexibility, the ability to manage competing priorities, and affordability, are challenging for young adults who are in insecure employment, facing financial instability, and dealing with social disadvantage (Kretsos 2010; Jaydarifard et al. 2023).

Research investigating patterns of dental attendance over time is primarily cross-sectional (Sanders et al. 2006; Roberts-Thomson et al. 2008; Martijn et al. 2017) or relies on self-reported historical patterns of attendance measured at 1 time point (Aldossary et al. 2015; Karimalakuzhiyil Alikutty and Bernabé 2016; Talakey and Bernabé 2019). Longitudinal evidence is needed to quantify dental attendance patterns over time. One longitudinal study from New Zealand has identified trajectories of dental visitation but only in 18- to 32-y-old New Zealand residents (Crocombe et al. 2011). There is no such evidence from Australia.

A comprehensive understanding of dental attendance over the life course can benefit from integrating both developmental and structural perspectives. Trajectory modeling are ideal for identifying groups of individuals with meaningfully different patterns (Nguena Nguefack et al. 2020). Modeling age- and time-based trajectories allows an exploration of health outcomes from a life course approach (Nagin 2014; Burton-Jeangros et al. 2015).Trajectory modeling is commonly used to explore developmental or age-related patterns, revealing how outcomes evolve across life stages. Age-trajectory analysis can provide insights into attendance patterns from young adulthood to retirement, reflecting key transitions such as entering the workforce or retiring (Crall and Forrest 2018). This aligns with the concept of life stages and transitions, which are fundamental to the developmental perspective (Burton-Jeangros et al. 2015; Crall and Forrest 2018). Time trajectory analysis incorporating sociodemographic factors such as income, education, and employment can show how structural factors shape health trajectories over time (Farmer et al. 2016; Jones et al. 2019). By identifying when individuals are most or least likely to seek dental care and identifying populations who require better health care, trajectory modeling has the potential to inform targeted health care policies (Nagin 2014; Burton-Jeangros et al. 2015; Nguena Nguefack et al. 2020).

We used trajectory modeling to identify (1) the age trajectories of dental attendance among Australian adults from young adulthood to retirement age and (2) if there have been distinct trajectories of dental attendance among Australian working-age adults over 8 y. To better understand the characteristics and inequalities associated with different dental attendance patterns over time, we compared the sociodemographic characteristics of individuals following different attendance trajectories.

## Methods

Two trajectory models were used to explore dental attendance: one based on age and the other based on time.

### Data Source

The Household, Income and Labour Dynamics in Australia (HILDA) study (Watson and Wooden 2012) began in 2001 and consists of a panel of about 17,000 Australians sampled annually. HILDA collects data about employment and the economic and personal well-being of individuals and households.

### Variables

#### Dental attendance (outcome)

Data on dental attendance are available for waves 9, 13, 17, and 21 of HILDA. COVID-19–related closures and physical distancing measures have been associated with a marked reduction in dental attendance (Patel 2020), so data from wave 21 (collected in 2021) were not used in the analysis. Respondents were asked to answer the following question: How long has it been since you last saw a dentist? Valid response options were (1) less than 6 mo ago, (2) 6 to 12 mo ago, (3) 1 to 2 y ago, (4) 2 to 5 y ago, (5) 5+ y ago, and (6) never been to a dentist. While there is no consensus on the optimal visit interval for oral health checks, once a year is a widely accepted interval (Fee et al. 2020). Clinical guidelines in the United Kingdom also recommend that this interval may be extended to a maximum of 2 y for patients demonstrating consistent oral health maintenance and low risk of oral disease (National Institute for Health and Care Excellence 2004). We categorized visits as frequent (less than 2 y ago) using survey response categories 1, 2, and 3 and as infrequent (2 y or longer) using categories 4, 5, and 6.

#### Descriptive variables

In addition to sex (male/female) and age (years), the following variables were used to describe the characteristics of participants. Remoteness location was categorized as major cities, inner regional, and outer regional/remote/very remote Australia. Highest education level attained was categorized into 3 categories: (1) bachelor or higher; (2) year 12, certificate or diploma; and (3) less than year 12. Employment was categorized as employed, unemployed, and not in the labor force. Weekly disposable income, which was defined as total household income after receipt of government benefits and deduction of income tax (Watson and Wooden 2012), was divided into tertiles. Disability was defined in HILDA as the presence or absence of any long-term health condition, impairment, or disability that restricts respondents in their everyday activities and has lasted or is likely to last for 6 mo or longer.

### Participant Selection

Although most Australians intend to retire after the age of 65 y when they become eligible for the age pension, the average age of retirement in Australia is 55 y (Australian Bureau of Statistics 2018–2019). Therefore, for examining age trajectories, age was restricted to 15 to 64 y, whereas for time trajectories, the analysis included individuals aged 24 to 54 y at baseline in 2009 (wave 9). For both trajectory models, only individuals with complete dental attendance data in wave 9 were included (*N* = 11,189 for age-based trajectory; *n* = 7,033 for time-based trajectory).

### Statistical Analysis

Group-based trajectory modeling (GBTM) was used to model age trajectories and time trajectories of dental attendance. First, age trajectories were fitted for individuals between 15 and 64 y of age using dental attendance data collected at 3 time points: 2009 (wave 9), 2013 (wave 13), and 2017 (wave 17). Next, the time trajectories of dental attendance were fitted using the 3 waves of data across the 8-y period from 2009 to 2017 (waves 9 to 17) for working-age adults.

#### GBTM

GBTM identifies latent subgroups within a population with homogenous patterns or trajectories of an outcome (Nagin 2005). GBTM classifies group membership of various trajectory groups based on sums of posterior probabilities of membership. The analysis was conducted using the user-written traj command (Jones and Nagin 2013) with a logit model in Stata version 17 (StataCorp 2021). For both age and time trajectory models, the following approach for model selection and model adequacy was used.

##### Model selection

A 2-stage model selection approach was used to determine the best-fitting model (Nagin 2005). In the first stage, we fitted models sequentially, starting with a 1-trajectory-group model up to a 5-group model, and for each model we assumed quadratic functions for the trajectory group. The model with the least negative value of Bayesian information criterion (BIC) and Akaike information criterion (AIC) was selected as the best fitting. In the second stage, the model shape was determined by specifying polynomial functions for the group selected from stage 1, and again, the best-fitting model was selected based on BIC, AIC, and the model adequacy criteria listed below.

##### Model adequacy

Model adequacy was also determined using the following criteria: (1) average posterior probability >70 for each trajectory group, (2) odds of correct classification >5.0 for each trajectory group, and (3) relative entropy close to 1 for each trajectory group (Hannah et al. 2018). In addition, we prioritized selecting the most parsimonious model that effectively captures distinctive and easily interpretable patterns (Nagin 2005).

#### Descriptive analysis

To quantify demographic and socioeconomic variations in dental attendance, we used the time trajectory model and fitted weighted univariate logistic regression models. The association between each descriptive (socioeconomic and demographic) variable at baseline in 2009 and the selected trajectory groups was estimated. We included descriptive variables based on the World Health Organization framework of social determinants of health (Solar and Irwin 2010), focusing on widely recognized indicators of socioeconomic position: income, education, and employment (Galobardes et al. 2006). In addition, we included remoteness and disability, which are associated with oral health inequalities in Australia (Australian Institute of Health and Welfare 2023).

The analysis is reported according to the Guidelines for Reporting on Latent Trajectory Studies (GRoLTS) (van de Schoot et al. 2017) (Appendix Table 1).The study received ethics approval from the Office of Research Ethics and Integrity, the University of Melbourne (reference No. 27248-43354-3).

## Results

This age trajectory analysis included 11,189 participants aged 15 to 64 y at baseline. The time trajectory analysis included 7,033 participants (i.e., a subset of all participants who were of working age). The characteristics of all participants (*N* = 11,189) in wave 9 (2009; baseline) are presented in Table 1. There was a similar proportion of males and females (48% vs. 52%). The median age of the participants was 38 y (interquartile range: 25–50 y). Most of the participants resided in major cities (62%) compared with inner regional (24%) and outer regional or remote areas (14%). Education levels varied for participants (23% had a bachelor or higher degree; 47% achieved year 12, certificate or diploma; and 31% has less than year 12). Of all participants, 75% were employed, and 23% identified as having a disability. The baseline characteristics of participants who were excluded are presented in Appendix Table 2. Of note is that males were more likely to have missing outcome data (attendance) compared with females (63% vs. 37%), while the proportions in those with attendance data were similar (48% vs. 52%).

**Table 1.** Participant Characteristics for Those Aged 15 to 64 y at Wave 9 (*N* = 11,189).

	*n*	%	95% CI
Median age (range), y^a^	38 (25–50)		
Age group (y)			
15 to 24	2,620	23.4	22.6–24.2
25 to 34	2,096	18.7	18.0–19.5
35 to 44	2,313	20.7	20.0–21.4
45 to 54	2,370	21.2	20.4–22.0
55 to 64	1,790	16.0	15.3–16.7
Sex			
Male	5,385	48.1	47.2–49.1
Female	5,804	51.9	51.0–52.8
Remoteness			
Major cities	6,945	62.1	61.2–63.0
Inner regional	2,713	24.3	23.5–25.1
Outer regional/remote/very remote	1,531	13.7	13.1–14.3
Education			
Bachelor or higher	2,549	22.8	22.0–23.6
Year 12, certificate, diploma	5,203	46.5	45.6–47.4
Less than year 12	3,433	30.7	29.8–31.6
Employment/labor force status			
Employed	8,373	74.8	74.0–75.6
Unemployed	494	4.4	4.1–4.8
Not in the labor force	2,322	20.8	20.0–21.5
Income (AUD)			
Tertile 1 (≥$1,949)	3,730	33.5	32.5–34.2
Tertile 2 ($1,313 to $1,948)	3,731	33.5	32.5–34.2
Tertile 3 (≤$1,312)	3,728	33.4	32.4–34.2
Disability/long-term health condition			
Yes	2,545	22.8	22.0–23.5
No	8,641	77.2	76.5–78.0

CI, confidence interval.

a Continuous age variable presented as median (interquartile range).

**Figure 1.**
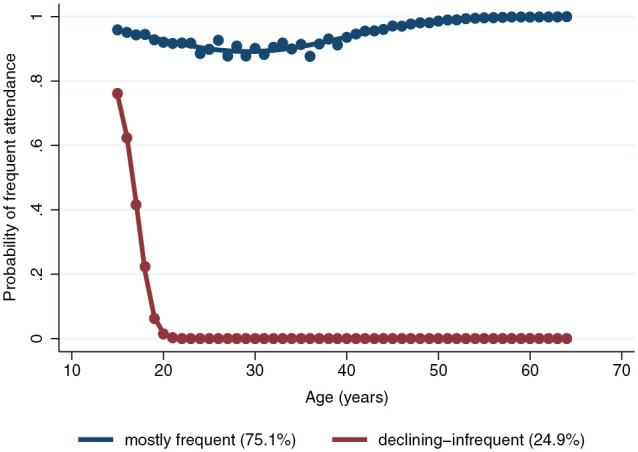
Age trajectories of dental attendance (2 group 2 2 model) (*N* = 11,189).

For the age trajectory model, 2 distinct trajectory groups of dental attendance emerged among participants aged 15 to 64 y: the mostly frequent and declining-infrequent group (Fig. 1). For the 11,189 participants, the group membership based on sums of posterior probabilities of being frequent dental attendees was 75.1% for the mostly frequent group and 24.9% for the declining-infrequent group. Almost all participants who were 15 y old at baseline had a high probability of being frequent attendees (Fig. 1). For the mostly frequent, there was a slight decline in the probability of regular attendance between ages 20 and 40 y, followed by an increase. For the declining-infrequent group, there was a sharp decline in the probability of being regular attendees between 15 and 20 y with no subsequent improvement.

**Figure 2.**
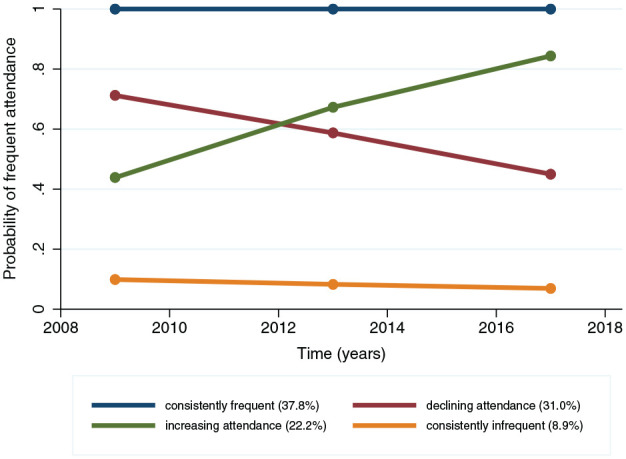
Time trajectories of dental attendance (4 group 1 1 1 1 model) (*n* = 7,033).

For the time-trajectory model, the 4-group model was selected as the best. As shown in Figure 2, for the working-age population (*n* = 7,033), group membership based on sums of posterior probabilities of being frequent dental attendees was highest for the consistently frequent group (37.8%) and lowest for the consistently infrequent group (8.9%). About 22% belonged to an increasing attendance trajectory: the increasing attendance group, whereas 31% exhibited a deterioration in attendance frequency: the declining attendance group. We note that none of the fitted time trajectory models fully met the adequacy criteria (see the notes in Appendix Tables 7 and 8). Therefore, following the adequacy criteria (Nagin 2005; Hannah et al. 2018), the most parsimonious model was selected.

The goodness-of-fit statistics for the selected model, model adequacy statistics, and alternate models that were tested are presented in Appendix Tables 3 to 5 (for age trajectories) and Appendix Tables 6 to 8 (time trajectories). The plots for the alternate models are presented in Appendix Figures 1 and 2.

To describe inequalities within time trajectories, each time trajectory group was compared to the consistently frequent group. As shown in Table 2, strong associations were seen between younger age groups, particularly those aged 25 to 34 y (odds ratio [OR]: 4.09, 95% confidence interval [CI]: 3.28–5.10) and 35 to 44 y (OR: 4.07, 95% CI: 3.28–5.05), and declining attendance. Females had a lower likelihood of declining (OR: 0.81, 95% CI: 0.70–0.95) or consistently infrequent attendance (OR: 0.48, 95% CI: 0.38–0.60), compared with the consistently frequent trajectory group. Overall, socioeconomic inequalities were evident, with measures such as education, employment, and income all showing that higher disadvantage was strongly associated with consistently infrequent dental attendance when compared with being consistently frequent. Compared with being frequent attendees, having a disability was associated with a slightly higher odds of being in the increasing attendance group (OR: 1.26, 95% CI: 1.04–1.52), but its association with declining or infrequent attendance was uncertain.

**Table 2.** Associations between Participant Characteristics at Wave 9 for Different Time Trajectory Attendance Groups.

	Attendance Trajectory Group		
Variable	Increasing versus Consistently FrequentOR (95% CI)^a^	Declining versus Consistently FrequentOR (95% CI)^a^	Consistently Infrequent versus Consistently FrequentOR (95% CI)^a^
Age group (y)			
45 to 54	Ref	Ref	Ref
35 to 44	1.13 (0.92–1.39)	4.07 (3.28–5.05)	1.76 (1.35–2.30)
24 to 34	1.35 (1.11–1.64)	4.09 (3.28–5.10)	1.75 (1.32–2.32)
Sex			
Male	Ref	Ref	Ref
Female	0.62 (0.53–0.73)	0.81 (0.70–0.95)	0.48 (0.38–0.60)
Remoteness			
Major cities	Ref	Ref	Ref
Inner regional	1.26 (1.05–1.51)	1.32 (1.10–1.58)	1.51 (1.18–1.93)
Outer regional/remote/very remote	1.38 (1.11–1.71)	1.26 (1.00–1.58)	1.25 (0.91–1.72)
Education			
Bachelor or higher	Ref	Ref	Ref
Year 12, certificate or diploma	1.77 (1.45–2.17)	1.68 (1.39–2.03)	2.37 (1.74–3.23)
Less than year 12	2.23 (1.77–2.82)	1.76 (1.40–2.20)	3.20 (2.24–4.56)
Employment status			
Employed	Ref	Ref	Ref
Unemployed	2.10 (1.39–3.16)	1.64 (1.05–2.56)	2.23 (1.28–3.88)
Not in the labor force	1.41 (1.12–1.78)	1.05 (0.83–1.32)	1.32 (0.99–1.76)
Weekly income			
Tertile 3 (≥$1,949)	Ref	Ref	Ref
Tertile 2 ($1,313 to $1,948)	1.50 (1.23–1.84)	1.73 (1.43–2.10)	1.84 (1.36–2.50)
Tertile 1 (≤$1,312)	1.83 (1.50–2.23)	1.81 (1.48–2.21)	2.05 (1.50–2.79)
Disability			
No	Ref	Ref	Ref
Yes	1.26 (1.04–1.52)	0.90 (0.74–1.09)	1.04 (0.80–1.34)

CI, confidence interval; OR, odds ratio.

a Estimated using survey weights.

## Discussion

This study aimed to document trajectories in dental attendance among Australian adults. For age-based trajectories, a clear decline in attendance during early adulthood was seen in both trajectory groups, and for about one-quarter of the population, this did not improve. Time-based trajectories identified 4 groups among working-age adults: 2 with changing attendance (increasing and declining) and 2 with stable attendance (consistently frequent and consistently infrequent). Inequalities were apparent in the time trajectories, with individuals facing educational, employment, and income disadvantages having a greater likelihood of being consistently infrequent attendees than individuals from socially advantaged groups. While people experiencing disabilities showed some improvement in dental attendance over time, they remained less likely to maintain consistent attendance compared with people without disabilities.

Our longitudinal findings complement prior cross-sectional studies that indicate a low level of dental attendance among Australians younger than 25 y (Roberts-Thomson and Stewart 2003; Slack-Smith et al. 2007). The observed decline in dental attendance could be due to the impact of dental costs among individuals aged 25 to 44 y (Duckett et al. 2019). This may also reflect the influence of Australian public policies, such as the low uptake and limited coverage provided by the Child Dental Benefits Schedule (CDBS), which ends at age 17 y, and the lack of equivalent support for young adults transitioning into adulthood (Moloney et al. 2024). However, it also important to acknowledge that developmental factors such as transition to working life and competing priorities and pressures during working life matter significantly. Young adults often face competing priorities such as employment insecurity, precarious employment, financial instability, and the pressure to succeed in a highly competitive job market (Kretsos 2010; Jaydarifard et al. 2023). These challenges cause stress and anxiety as individuals juggle professional aspirations and personal responsibilities (Jaydarifard et al. 2023). Experiencing social disadvantage can further exacerbate these pressures, especially with inflation, making it even harder to prioritize their health and well-being. Our results agree with a New Zealand study (Crocombe et al. 2011), which found a decline in routine attendance among young working-age adults. While Crocombe et al. (2011) showed a gradual decline in attendance leading to lower attendance levels at 26 y, our findings showed a sudden decrease in attendance between ages 15 and 20 y. This dissimilarity may reflect differences in structural factors such as health care systems and/or support policies between New Zealand and Australia.

Individuals facing socioeconomic disadvantage are more likely to exhibit consistently infrequent dental attendance over the long term. This highlights the need to address persistent structural barriers affecting dental attendance over time through equitable policies.

Overall, the drop in dental attendance coinciding with critical life changes and the loss of CDBS protection necessitates research on their effects on dental attendance. Notably, our study does not establish a causal relationship between socioeconomic characteristics and attendance trajectories but rather shows socioeconomic and demographic variations in the composition of time-based trajectory groups. This aligns with several previous studies that have consistently linked dental attendance with socioeconomic position (Reda, Reda, et al. 2018).

We note some key strengths. First, by using nationally representative longitudinal data from HILDA, we reported how dental attendance patterns in Australia have changed over time—an advantage over cross-sectional studies. Second, we included both age- and time-based trajectories to provide a comprehensive understanding of dental attendance dynamics using a life course approach. Third, we used GBTM, in which each observation is associated with a set of probabilities, indicating the likelihood of the observation belonging to each trajectory group (Nguena Nguefack et al. 2020). This approach acknowledges that people’s long-term dental attendance behaviors may not conform neatly to a single category. However, due to the absence of information on whether participants visited the same dentist and the purpose of the visits (symptomatic or preventive), it was not feasible to obtain a comprehensive understanding of favorable and unfavorable patterns of attendance. While this is a limitation of our study, it establishes the need for better longitudinal oral health data in Australia. In addition, descriptive analysis was conducted for time trajectories but not age trajectories. Different measures of social position affect the life course differently, and their time-varying nature further complicates the quantification of their composition within age trajectories. The exclusion of participants missing dental attendance during the first wave of outcome measurement (wave 9) might lead to selection bias. However, they were excluded because, with only 3 time points of outcome available, including them could compromise the accuracy of the GBTM model. Due to limited waves of data, our analysis is limited by small-group populations. Also, having only 3 time points in our study did not allow fitting higher-order polynomial functions (>2) for the time trajectory model. Due to these data limitations, even the best-fitting time-trajectory model did not fulfill all model adequacy criteria (Appendix Table 8).

The identified trajectories have important policy implications. Preventive health care policies must be informed by the dynamics of life stages and their effects on attendance behaviors, rather than focusing solely on specific ages. Sustained measures addressing barriers such as affordability and waiting times are needed. Future research should explore how dynamic changes in socioeconomic position over time influence dental attendance trajectories, building on the baseline analysis presented in this study. Future research could also explore the mechanisms underlying specific trajectories, considering factors such as socioeconomic status and access to oral health care services, particularly among those with declining and consistently low attendance. Attendance patterns among individuals with disabilities require further investigation, including identifying factors that support sustained improvements in attendance and ensuring consistency over time. Furthermore, barriers and facilitators that influence dental attendance within this population need to be explored.

## Conclusion

Our findings provide critical insights into the patterning of dental attendance patterns over time among Australian adults throughout adulthood. The identification of distinct patterns over time and across age groups adds valuable information for understanding long-term dental attendance behavior.

## Author Contributions

G. Kaur, contributed to conception, design, data acquisition, analysis, and interpretation, drafted and critically revised the manuscript; T. King, A. Karahalios, contributed to design, data analysis and interpretation, critically revised the manuscript; A. Singh, contributed to conception and design, data acquisition, analysis, and interpretation, critically revised the manuscript. All authors gave final approval and agree to be accountable for all aspects of the work.

## Supplemental Material

sj-docx-1-jdr-10.1177_00220345251315155 – Supplemental material for Longitudinal Trajectories of Dental Attendance in Australian Adults Supplemental material, sj-docx-1-jdr-10.1177_00220345251315155 for Longitudinal Trajectories of Dental Attendance in Australian Adults by G. Kaur, T. King, A. Karahalios and A. Singh in Journal of Dental Research

## References

[bibr1-00220345251315155] Aldossary A Harrison VE Bernabé E. 2015. Long-term patterns of dental attendance and caries experience among British adults: a retrospective analysis. Eur J Oral Sci. 123(1):39–45. 25521216 10.1111/eos.12161

[bibr2-00220345251315155] Australian Bureau of Statistics. 2018–2019. Retirement and retirement intentions, Australia. Canberra: Australian Bureau of Statistics; [accessed 2025 Jan 8]. https://www.abs.gov.au/statistics/labour/employment-and-unemployment/retirement-and-retirement-intentions-australia/2018-19.

[bibr3-00220345251315155] Australian Institute of Health and Welfare. 2023. Oral health and dental care in Australia. Canberra: Australian Institute of Health and Welfare; [accessed 2025 Jan 8]. https://www.aihw.gov.au/getmedia/fb0d5c81-7661-4bb3-a049-121c7e81c021/oral-health-and-dental-care-in-australia-tranche-7-21-november-2023.pdf.

[bibr4-00220345251315155] Burton-Jeangros C Cullati S Sacker A Blane D. 2015. A life course perspective on health trajectories and transitions. Cham (Swizterland): Springer. 27683923

[bibr5-00220345251315155] Crall JJ Forrest CB . 2018. A life course health development perspective on oral health. In: Halfon N Forrest CB Lerner RM Faustman EM , editors. Handbook of life course health development. Cham (Switzerland): Springer. p. 299–320. 31314282

[bibr6-00220345251315155] Crocombe LA Broadbent JM Thomson WM Brennan DS Slade GD Poulton R. 2011. Dental visiting trajectory patterns and their antecedents. J Public Health Dent. 71(1):23–31. 20880031 10.1111/j.1752-7325.2010.00196.x

[bibr7-00220345251315155] Duckett S Cowgill M Swerissen H. 2019. Filling the gap: a universal dental scheme for Australia. Melbourne (Australia): Grattan Institute; [accessed 2025 Jan 8]. https://grattan.edu.au/report/filling-the-gap/.

[bibr8-00220345251315155] Ellershaw AC Spencer AJ ; Australian Institute of Health and Welfare. 2011. Dental attendance patterns and oral health status. Canberra: Australian Institute of Health and Welfare; [accessed 2025 Jan 8]. https://www.aihw.gov.au/getmedia/e83cc818-5c2d-4827-8808-235822cad0bc/12910.pdf?v=20230605171925&inline=true.

[bibr9-00220345251315155] Farmer J McLeod L Siddiqi A Ravaghi V Quiñonez C. 2016. Towards an understanding of the structural determinants of oral health inequalities: a comparative analysis between Canada and the United States. SSM Popul Health. 2:226–236. 29349142 10.1016/j.ssmph.2016.03.009 PMC5757973

[bibr10-00220345251315155] Fee PA Riley P Worthington HV Clarkson JE Boyers D Beirne PV . 2020. Recall intervals for oral health in primary care patients. Cochrane Database Syst Rev. 10(10):CD004346. doi:10.1002/14651858.CD004346.pub5 PMC8256238 33053198

[bibr11-00220345251315155] Galobardes B Shaw M Lawlor DA Lynch JW Davey Smith G. 2006. Indicators of socioeconomic position (part 1). J Epidemiol Community Health. 60(1):7–12. 10.1136/jech.2004.023531 PMC2465546 16361448

[bibr12-00220345251315155] Hannah L Scott K Matthew S Iain B Amanda JC Michael L Michael BC Andrew GR . 2018. Framework to construct and interpret latent class trajectory modelling. BMJ Open. 8(7):e020683. doi:10.1136/bmjopen-2017- 020683 10.1136/bmjopen-2017-020683 PMC6042544 29982203

[bibr13-00220345251315155] Jaydarifard S Smith SS Mann D Rossa KR Nikooharf Salehi E Gnani Srinivasan A Shekari Soleimanloo S. 2023. Precarious employment and associated health and social consequences; a systematic review. Aust N Z J Public Health. 47(4):100074. doi:10.1016/j.anzjph.2023.100074 37453888

[bibr14-00220345251315155] Jones BL Nagin DS . 2013. A note on a Stata plugin for estimating group-based trajectory models. Sociol Methods Res. 42(4):608–613.

[bibr15-00220345251315155] Jones NL Gilman SE Cheng TL Drury SS Hill CV Geronimus AT . 2019. Life course approaches to the causes of health disparities. Am J Public Health. 109(S1):S48–S55. 10.2105/AJPH.2018.304738 PMC6356123 30699022

[bibr16-00220345251315155] Karimalakuzhiyil Alikutty F Bernabé E . 2016. Long-term regular dental attendance and periodontal disease in the 1998 adult dental health survey. J Clin Periodontol. 43(2):114–120. 26932321 10.1111/jcpe.12496

[bibr17-00220345251315155] Kretsos L . 2010. The persistent pandemic of precariousness: young people at work. In: Tremmel J , editor. A young generation under pressure? Heidelberg (Germany): Springer Berlin. p. 3–21.

[bibr18-00220345251315155] Martijn JL Jacques SNV Luc CM Luc MJDV . 2017. Socioeconomic inequalities in caries experience, care level and dental attendance in primary school children in Belgium: a cross-sectional survey. BMJ Open. 7(7):e015042. doi:10.1136/bmjopen-2016-015042 PMC5541598 28729310

[bibr19-00220345251315155] Moloney G Amos K Edser S Barone C. 2024. Socially constructed beliefs and the uptake of the Child Dental Benefits Schedule. Aust Dent J. 69(3):197–205. 38523271 10.1111/adj.13015

[bibr20-00220345251315155] Nagin DS. 2005. Group-based modeling of development. Cambridge (MA): Harvard University Press.

[bibr21-00220345251315155] Nagin DS . 2014. Group-based trajectory modeling: an overview. Ann Nutr Metab. 65(2–3):205–210. 25413659 10.1159/000360229

[bibr22-00220345251315155] National Institute for Health and Care Excellence. 2004. Dental recall: recall interval between routine dental examinations. National Institute for Health and Care Excellence: guidelines. London: National Collaborating Centre for Acute Care (UK). 21678631

[bibr23-00220345251315155] Nguena Nguefack HL Pagé MG Katz J Choinière M Vanasse A Dorais M Samb OM Lacasse A. 2020. Trajectory modelling techniques useful to epidemiological research: a comparative narrative review of approaches. Clin Epidemiol. 12:1205–1222. 33154677 10.2147/CLEP.S265287 PMC7608582

[bibr24-00220345251315155] Patel N . 2020. Impact on dental economics and dental healthcare utilization in COVID-19: an exploratory study. J Adv Oral Res. 11(2):128–136.

[bibr25-00220345251315155] Reda SF Reda SM Thomson WM Schwendicke F. 2018. Inequality in utilization of dental services: a systematic review and meta-analysis. Am J Public Health. 108(2):e1–e7. doi:10.2105/AJPH.2017.304180 PMC5846590 29267052

[bibr26-00220345251315155] Reda SM Krois J Reda SF Thomson WM Schwendicke F. 2018. The impact of demographic, health-related and social factors on dental services utilization: systematic review and meta-analysis. J Dent. 75:1–6. 29673686 10.1016/j.jdent.2018.04.010

[bibr27-00220345251315155] Roberts-Thomson KF Luzzi L Brennan DS . 2008. Social inequality in use of dental services: relief of pain and extractions. Aust N Z J Public Health. 32(5):444–449. 18959548 10.1111/j.1753-6405.2008.00277.x

[bibr28-00220345251315155] Roberts-Thomson KF Stewart JF . 2003. Access to dental care by young South Australian adults. Aust Dent J. 48(3):169–174. 14640369 10.1111/j.1834-7819.2003.tb00027.x

[bibr29-00220345251315155] Sanders AE Spencer AJ Slade GD . 2006. Evaluating the role of dental behaviour in oral health inequalities. Community Dent Oral Epidemiol. 34(1):71–79. 16423034 10.1111/j.1600-0528.2006.00261.x

[bibr30-00220345251315155] Slack-Smith LM Mills CR Bulsara MK O’Grady MJ . 2007. Demographic, health and lifestyle factors associated with dental service attendance by young adults. Aust Dent J. 52(3):205–209. 17969289 10.1111/j.1834-7819.2007.tb00490.x

[bibr31-00220345251315155] Solar O Irwin A. 2010. A conceptual framework for action on the social determinants of health. Geneva: World Health Organization; [accessed 2025 Jan 8]. https://www.who.int/publications/i/item/9789241500852.

[bibr32-00220345251315155] StataCorp. 2021. Stata statistical software: release 17. College Station (TX): StataCorp LLC.

[bibr33-00220345251315155] Talakey AA Bernabé E. 2019. Long-term regular dental attendance and tooth retention among British adults: a cross-sectional analysis of national survey data. Int J Dent Hyg. 17(1):64–70. 30381874 10.1111/idh.12373

[bibr34-00220345251315155] Thomson WM Williams SM Broadbent JM Poulton R Locker D. 2010. Long-term dental visiting patterns and adult oral health. J Dent Res. 89(3):307–311. 20093674 10.1177/0022034509356779 PMC2821461

[bibr35-00220345251315155] van de Schoot R Sijbrandij M Winter SD Depaoli S Vermunt JK . 2017. The GRoLTS-Checklist: guidelines for reporting on latent trajectory studies. Structural Equation Modeling. 24(3):451–467.

[bibr36-00220345251315155] Watson N Wooden M. 2012. The HILDA survey: a case study in the design and development of a successful household panel study. Longitudinal and Life Course Studies. 3(3):369–381.

[bibr37-00220345251315155] World Health Organization. 2022. Global oral health status report: towards universal health coverage for oral health by 2030. Geneva: World Health Organization; [accessed 2025 Jan 8]. https://www.who.int/publications/i/item/9789240061484.

