# Supplementary material for: Longitudinal Trajectories of Dental Attendance in Australian Adults
Source: J Dent Res. 2025 Mar 12;104(6):604–10. doi: 10.1177/00220345251315155 (PMC12075884; doi:10.1177/00220345251315155)
Supplement: sj-docx-1-jdr-10.1177_00220345251315155 – Supplemental material for Longitudinal Trajectories of Dental Attendance in Australian Adults [file sj-docx-1-jdr-10.1177_00220345251315155.docx]

# Supplementary appendix

Appendix table1: Guidelines for Reporting on Latent Trajectory Studies (GRoLTS) checklist checklist

| 1 | Is the metric of time used in the statistical model reported? | Methods |
| --- | --- | --- |
| 2 | Is information presented about the mean and variance of time within a wave? | Methods |
| 3a. | Is the missing data mechanism reported? | Methods |
| 3b. | Is a description provided of what variables are related to attrition/missing data? | Methods; Appendix table 2 |
| 3c. | Is a description provided of how missing data in the analyses were dealt with? | Methods |
| 4 | Is information about the distribution of the observed variables included? | Methods; Results; Appendix table 2 |
| 5 | Is the software mentioned? | Methods |
| 6a. | Are alternative specifications of within-class heterogeneity considered (e.g., LGCA vs. LGMM) and clearly documented? If not, was sufficient justification provided as to eliminate certain specifications from consideration? | Methods |
| 6b. | Are alternative specifications of the between-class differences in variance “covariance matrix structure considered and clearly documented? If not, was sufficient justification provided as to eliminate certain specifications from consideration? | Methods |
| 7 | Are alternative shape/functional forms of the trajectories described? | Appendix tables 4 & 7 |
| 8 | If covariates have been used, can analyses still be replicated? | Not relevant |
| 9 | Is information reported about the number of random start values and final iterations included? | Not relevant |
| 10 | Are the model comparison (and selection) tools described from a statistical perspective? | Methods; Appendix tables 3-4 & 6-7 |
| 11 | Are the total number of fitted models reported, including a one-class solution? | Appendix tables 3-4 & 6-7 |
| 12 | Are the number of cases per class reported for each model (absolute sample size, or proportion)? | Appendix tables 5 & 8 |
| 13 | If classification of cases in a trajectory is the goal, is entropy reported? | Appendix tables 3-4 & 6-7 |
| 14a. | Is a plot included with the estimated mean trajectories of the final solution? | Results Figure1 & 2 |
| 14b. | Are plots included with the estimated mean trajectories for each model? | Appendix figures 1 and 2 |
| 14c. | Is a plot included of the combination of estimated means of the final model and the observed individual trajectories split out for each latent class? | Appendix figures 3a-b |
| 15 | Are characteristics of the final class solution numerically described (i.e., means, SD/SE, n, CI, etc.)? | Appendix tables 5 & 8 |
| 16 | Are the syntax files available (either in the appendix, supplementary materials, or from the authors)? | Available from authors upon request |

Appendix table2: Baseline characteristics of included and excluded participants

|  | Dental attendance data recorded for wave 9 | | Excluded due to missing dental attendance in wave 9 | |
| --- | --- | --- | --- | --- |
|  | n | % | n | % |
| Age group (years) |  |  |  |  |
| 18-24 | 2,620 | 23.40 | 172 | 27.20 |
| 25-34 | 2,096 | 18.70 | 116 | 18.30 |
| 35-44 | 2,313 | 20.70 | 95 | 15.00 |
| 45-54 | 2,370 | 21.20 | 151 | 23.90 |
| 55-64 | 1,790 | 16.00 | 99 | 15.60 |
| Sex |  |  |  |  |
| Male | 5,385 | 48.10 | 399 | 63.00 |
| Female | 5,804 | 51.90 | 234 | 37.00 |
| Remoteness |  |  |  |  |
| Major cities | 6,945 | 62.10 | 396 | 62.60 |
| Inner regional | 2,713 | 24.20 | 138 | 21.80 |
| Outer regional/remote/very remote | 1,531 | 13.70 | 99 | 15.60 |
| Education |  |  |  |  |
| Bachelor or higher | 2,549 | 22.80 | 2 | 14.30 |
| Year 12, certificate, diploma | 5,203 | 46.50 | 8 | 57.10 |
| Less than year 12 | 3,433 | 30.70 | 4 | 28.60 |
| Employment/labour force status |  |  |  |  |
| Missing data | 0 | 0.00 | 619 | 97.80 |
| Employed | 8,373 | 74.80 | 7 | 1.10 |
| Unemployed | 494 | 4.40 | 0 | 0.00 |
| Not in the labour force | 2,322 | 20.80 | 7 | 1.10 |
| Weekly Income |  |  |  |  |
| Tertile1 | 3,786 | 33.80 | 155 | 24.50 |
| Tertile2 | 3,730 | 33.30 | 213 | 33.60 |
| Tertile3 | 3,673 | 32.80 | 265 | 41.90 |
| Disability/ Long term health condition |  |  |  |  |
| Missing data | 0 | 0.00 | 619 | 98.30 |
| Yes | 2,545 | 22.80 | 5 | 0.80 |
| No | 8,641 | 77.20 | 6 | 1.00 |

Appendix tables 3-5 contain statistics for age trajectory models of dental attendance

Appendix table 3: Goodness of fit statistics for models with 1-5 age-based trajectory groups

| **Number of groups** | **Polynomial function** | **BIC** | **AIC** | **Entropy** |
| --- | --- | --- | --- | --- |
| **1** | 2 | -6584.87 | -6573.89 | - |
| **2** | **2 2** | **-6552.34** | **-6526.71** | **0.80** |
| **3** | 2 2 2 | -6617.58 | -6577.31 | 0.20 |
| 4 | 2 2 2 2 | -6585.24 | -6530.32 | 0.47 |
| **5** | 2 2 2 2 2 | -6607.04 | -6537.47 | 0.28 |

Note: This table presents the key goodness of fit statistics for models with 1-5 trajectory groups using quadratic polynomial distribution for each (i.e., polynomial function = 2). The 2-group model was selected as the best fitting model as it has the highest (least negative) BIC and AIC values. After this stage, the shape of the model was determined by specifying different polynomial functions for the selected 2 group model.

Appendix table 4: Statistics for 2 group age-based trajectory models with different polynomial functions

| **Number of groups** | **Polynomial function** | **BIC** | **AIC** | **Entropy** |
| --- | --- | --- | --- | --- |
| **2** | **2 2** | **-6552.34** | **-6526.71** | **0.80** |
| 2 | 3 3 | -6559.23 | -6526.28 | 0.77 |
| 2 | 1 2 | -6570.48 | -6548.51 | 0.95 |
| 2 | 2 3 | -6578.79 | -6549.50 | 0.92 |
| 2 | 3 2 | -6579.59 | -6550.30 | 0.92 |

Note: Table shows the top five models according to BIC. The 2 group quadratic (2 2) model was selected as the final model.

Appendix table 5: Characteristics of trajectory membership for the selected age-based model i.e., 2 group quadratic model.

|  | Group 1  *mostly frequent* | Group 2  *declining infrequent* |
| --- | --- | --- |
| Group membership (%) | 75.1 | 24.9 |
| Average posterior probability (%) | 98.30 | 85.48 |
| Odds of correct classification | 19.11 | 17.80 |
| Count per group | 8097 | 3092 |

Appendix tables 6-8 contain statistics for time trajectory models of dental attendance

Appendix table 6: Goodness of fit statistics for models with 1-5 time-based trajectory groups

| **Number of groups** | **Polynomial function** | **BIC** | **AIC** | **Entropy** |
| --- | --- | --- | --- | --- |
| **1** | 2 | -9837.02 | -9825.47 | - |
| **2** | 2 2 | -9309.52 | -9282.57 | 0.24 |
| **3** | 2 2 2 | -9294.16 | -9251.81 | 0.34 |
| **4** | **2 2 2 2** | **-9289.72** | **-9231.97** | **0.35** |
| **5** | 2 2 2 2 2 | -9337.98 | -9264.84 | 0.26 |

Note: This table presents the key goodness of fit statistics for models with 1-5 trajectory groups using quadratic polynomial distribution (polynomial function = 2) for each. The 4-group model was selected as the best fit model as it has the highest (least negative) BIC and AIC values. After this stage, the shape of the model was determined by specifying different polynomial functions for the selected 4 group model.

Appendix table 7: Statistics for 4 group time-based models with different polynomial functions

| **Number of groups** | **Polynomial function** | **BIC** | **AIC** | **Entropy** |
| --- | --- | --- | --- | --- |
| **4** | **1111** | **-9270.32** | **-9227.97** | **0.42** |
| 4 | 1112 | -9276.29 | -9230.09 | 0.50 |
| 4 | 1221 | -9280.02 | -9229.97 | 0.65 |
| 4 | 2121 | -9280.02 | -9229.97 | 0.50 |
| 4 | 1212 | -9280.26 | -9230.21 | 0.63 |

Note: Table shows the top five models according to BIC. Even though the 2^nd^ and the 3^rd^ models had similar BIC and better entropy than the first, the first model was selected as the best fit because the other models resulted in some groups having very low membership (<5%).

Appendix table 8: Characteristics of trajectory membership for the selected model i.e., 4 group linear model.

|  | Group 1  *consistently frequent* | Group 2  *decreasing attendance* | Group 3  *Improving attendance* | Group 4  *consistently infrequent* |
| --- | --- | --- | --- | --- |
| Group membership (%) | 37.8 | 31.0 | 22.2 | 8.9 |
| Average posterior probability (%) | 67.54* | 72.64 | 57.51* | 64.13* |
| Odds of correct classification | 3.42^#^ | 5.90 | 4.73^#^ | 18.24 |
| Count per group | 3936 | 1220 | 1301 | 576 |

Note: *The average posterior probabilities in three out of four groups were lower than ideal (>70)

#The odds of correct classification in two groups were less than the recommended value (>5.0)

Appendix figures 1a – 1d contain plots for age trajectory models of dental attendance


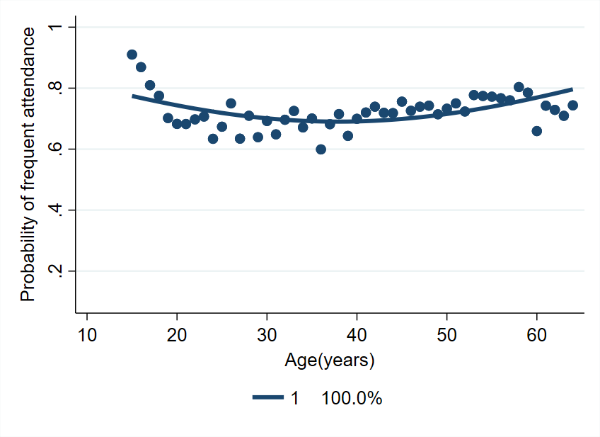


Appendix figure 1a. One group age trajectory model


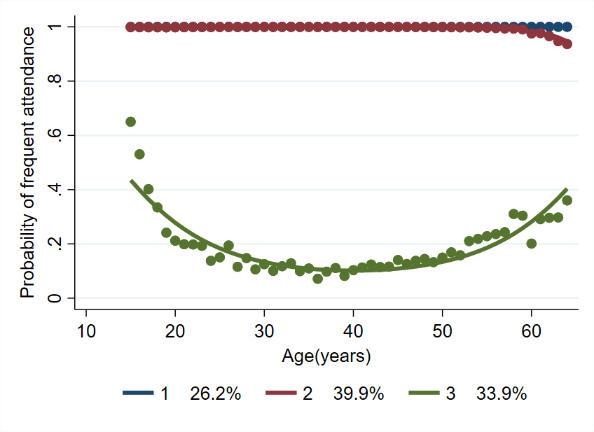


Appendix figure 1b. Three group age trajectory model


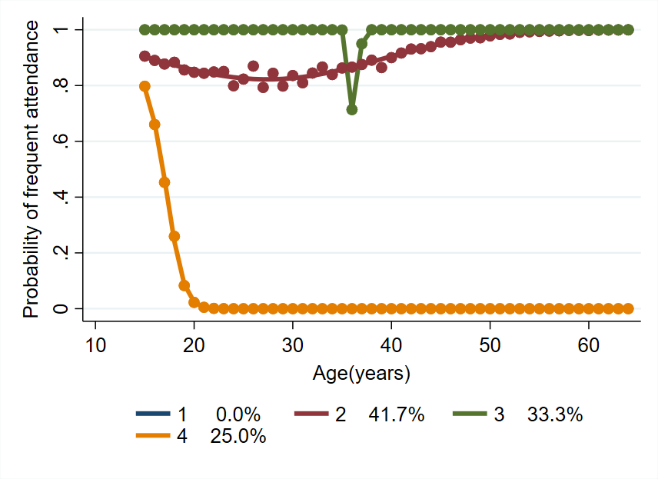


Appendix figure 1c. Four group age trajectory model


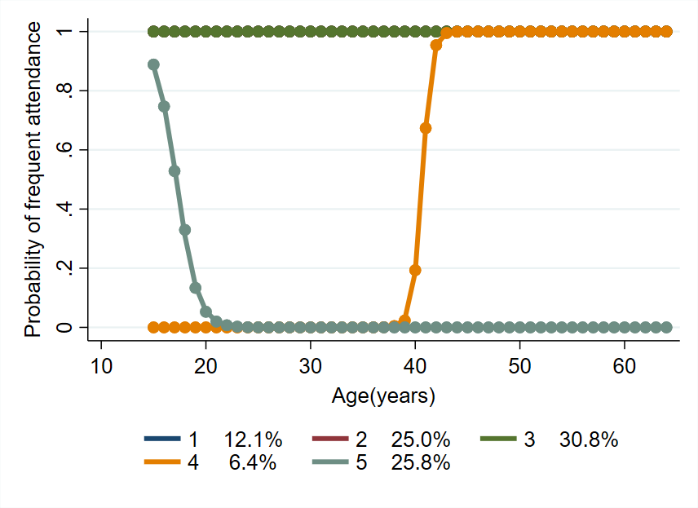


Appendix figure 1d. Five group age trajectory model

Appendix figures 2a – 2d contain plots for time trajectory models of dental attendance


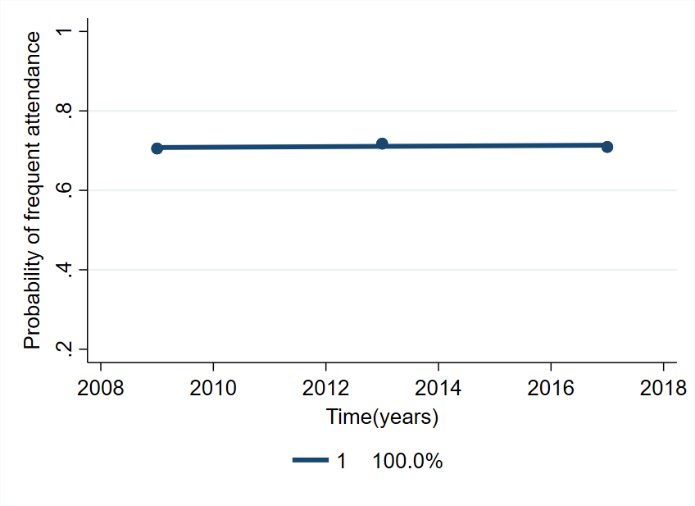


Appendix figure 2a. One group time trajectory model


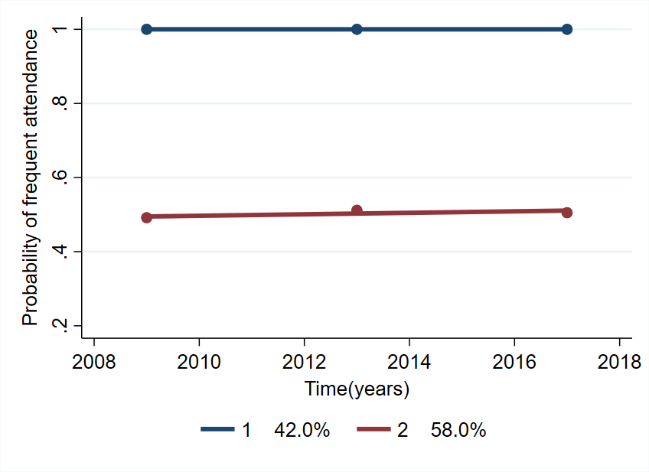


Appendix figure 2b. Two group time trajectory model


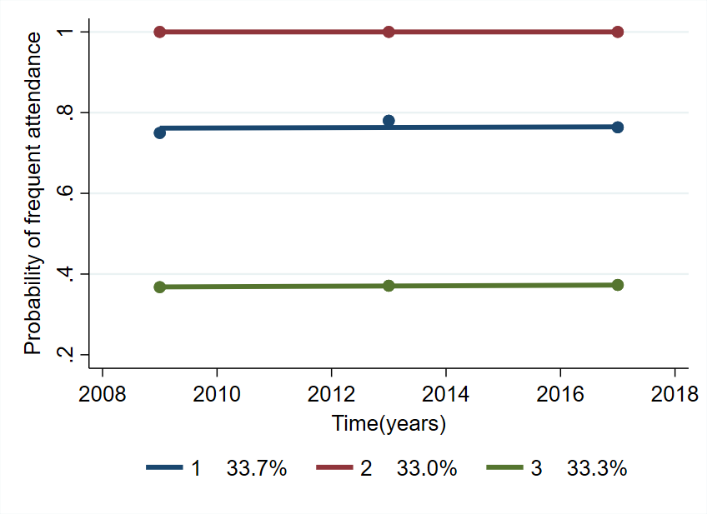


Appendix figure 2c. Three group time trajectory model


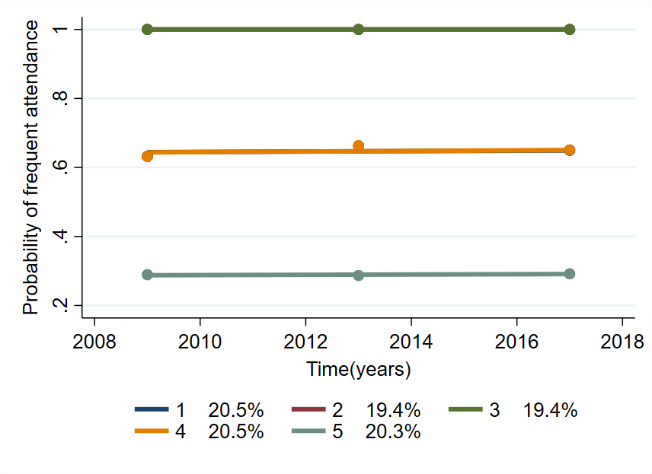


Appendix figure 2d. Five group time trajectory model

Appendix figures 3a & 3b contain plots of the estimated and observed mean probabilities of the final age and time trajectory models.


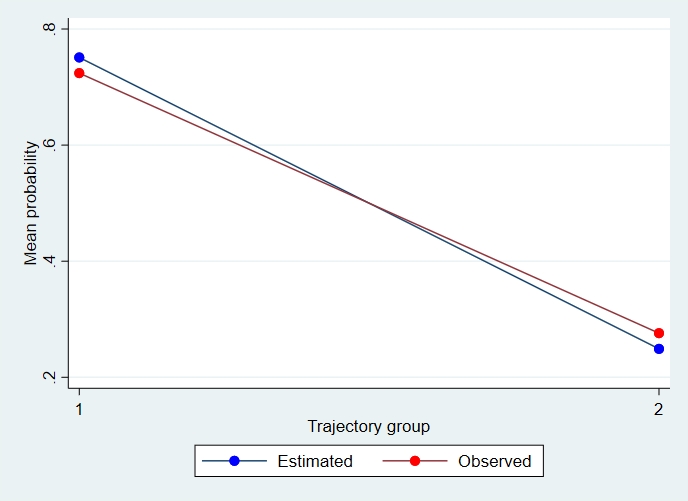


Appendix figure 3a. Plots of the estimated and observed probabilities of the final age model for each trajectory group


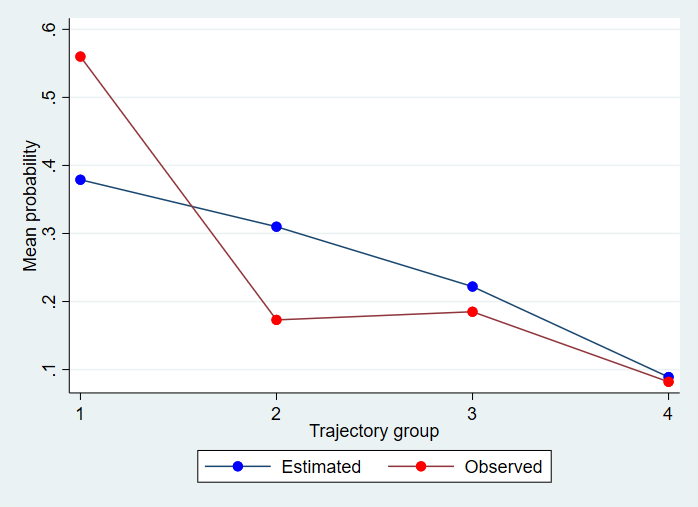


Appendix figure 3b. Plots of the estimated and observed probabilities of the final time model for each trajectory group
